# Linear and non-linear Mendelian randomization analyses of sex-specific associations between sleep duration and hyperuricemia

**DOI:** 10.3389/fnut.2022.920791

**Published:** 2022-10-10

**Authors:** Chenfeng Zou, Zhenqian Wang, Wenyu Huang, Jiawen Lu, Vivian Yawei Guo, Yuying Zhang, Shufei Zang, Jinying Yang, Liyuan Han, Guozhi Jiang

**Affiliations:** ^1^School of Public Health (Shenzhen), Sun Yat-sen University, Shenzhen, Guangdong, China; ^2^Department of Epidemiology, School of Public Health, Sun Yat-sen University, Guangzhou, China; ^3^Shenzhen Longhua Maternity and Child Healthcare Hospital, Shenzhen, China; ^4^Department of Endocrinology, Shanghai Fifth People’s Hospital, Fudan University, Shanghai, China; ^5^Department of Obstetrics, Longgang District Maternal & Child Healthcare Hospital of Shenzhen City, Shenzhen, China; ^6^Department of Global Health, Ningbo Institute of Life and Health Industry, University of Chinese Academy of Sciences, Ningbo, Zhejiang, China

**Keywords:** sleep duration, urate, hyperuricemia, Mendelian randomization, non-linear

## Abstract

**Background:**

Observational studies have suggested a potential non-linear association between sleep duration and hyperuricemia. However, the causal nature and sex-specific differences are poorly understood. We aimed to determine the shape of sex-specific causal associations between sleep duration and hyperuricemia in the UK Biobank.

**Methods:**

Logistic regression was used to investigate the observational association between self-reported sleep duration and hyperuricemia among 387,980 white British participants (mean age: 56.9 years and 46.0% males). Linear and non-linear Mendelian Randomization (MR) analyses were performed to assess the causal association between continuous sleep duration and hyperuricemia. The causal effects of genetically predicted short (<7 h) and long (>8 h) sleep durations on hyperuricemia were further estimated, respectively.

**Results:**

Traditional observational analysis suggested U- and J-shaped associations between sleep duration and hyperuricemia in females and males, respectively. Linear MR did not support the causal effect of sleep duration on hyperuricemia. Non-linear MR demonstrated an approximately U-shaped causal association between continuous sleep duration and hyperuricemia in overall participants and females, but not in males. Genetically predicted short sleep duration was significantly associated with hyperuricemia in females (OR [95% CI]: 1.21 [1.08–1.36]; *P* = 0.001), but not in males (1.08 [0.98–1.18]; *P* = 0.137). By contrast, genetically predicted long sleep duration was not significantly associated with the risk of hyperuricemia in either females or males.

**Conclusion:**

Genetically predicted short sleep duration is a potential causal risk factor for hyperuricemia for females but has little effect on males. Long sleep duration does not appear to be causally associated with hyperuricemia.

## Introduction

Hyperuricemia is a common metabolic disease mainly caused by disorders of purine metabolism (1). With an ascending trend in the prevalence of hyperuricemia worldwide (2, 3), it has been considered one of the major public health concerns in recent years. According to a nationally representative survey of the United States, approximately 22.8 million males and 24.4 million females met the sex-specific criteria for hyperuricemia in 2015–2016, corresponding to an annual prevalence of around 20% (3). Furthermore, evidence has demonstrated that hyperuricemia is not only a pre-requisite for gout (4) but also associated with a series of chronic diseases, including metabolic syndrome (5), diabetes mellitus (6), as well as chronic kidney disease (7). So far, however, the underlying pathological mechanism of hyperuricemia has not been completely elucidated, and long-term urate-lowering therapy carries both risks and costs (8, 9). For example, long-term use of urate-lowering medications (e.g., febuxostat) would not only contribute to tremendous healthcare costs but also raise concerns about potential higher all-cause and cardiovascular mortality (8). Thus, it is urgent and significant to identify modifiable hyperuricemia-related risk factors and initiate early prevention to reduce the burden of its potentially associated detrimental outcome on health.

Optimal sleep duration plays an essential role in maintaining human health and wellbeing (10). Previous studies have identified extreme sleep duration as a critical lifestyle risk factor for a series of chronic diseases (11), such as diabetes mellitus (12) and chronic kidney disease (13, 14). Recently, several studies have suggested that sleep duration may be a potentially modifiable risk factor for hyperuricemia (15–18). Interestingly, one of those studies based on data from the China Health and Nutrition Survey (CHNS) found a significant association between sleep duration and hyperuricemia only in females but not in males (16). That said, the association between sleep duration and hyperuricemia may be sex-specific.

Furthermore, another study conducted with 6,151 Korean women showed a significant U-shaped association between sleep duration and hyperuricemia (18). A recent study also reported the non-linear associations between sleep duration and overall health status (19). However, there is still a paucity of data on the sex-specific shape of associations between sleep duration and hyperuricemia in white populations. Moreover, the findings from conventional observational studies, even well-designed studies with large sample sizes, are prone to be biased by unknown confounding factors and reverse causation (20, 21). Therefore, a better understanding of the true shape of sex-specific associations between sleep duration and hyperuricemia may shed light on the prevention and intervention strategies aimed at establishing the reference range of sleep duration and, consequently, reducing the risk of hyperuricemia.

Unlike conventional observational analyses, Mendelian randomization (MR) analyses have the advantageous applicability of making causal inferences using genetic variants, which are randomly distributed at conception (22), as instrumental variables for modifiable exposures to assess the potential causal relationship with outcomes (23). Thus, MR is naturally immune to the limitations of residual confounding and reverse causation in observational studies (20, 21). To the best of our knowledge, no study has attempted to investigate the causal associations between sleep duration and hyperuricemia. Moreover, as standard MR is based on the assumption of linearity (24), it would overlook non-linear effects (25). Given that a U-shaped observational association between sleep duration and hyperuricemia was reported in a previous study (18), it is a key research priority to explore the potential non-linear causal relationship between sleep duration and hyperuricemia.

In the present study, we aimed to determine the shape of associations between sleep duration and hyperuricemia in the UK Biobank. We hypothesized in advance that the associations were non-linear and sex-specific. To confirm the hypothesis, the associations between sleep duration and hyperuricemia would be investigated not only in overall participants, but also in males and females. First, we performed cross-sectional analyses to investigate the shape and strength of the observational associations between sleep duration and hyperuricemia. Then, we conducted linear MR analyses to determine the linear causal associations between genetically predicted continuous sleep duration and hyperuricemia. We further characterized the shape of the causal associations using a state-of-the-art non-linear MR approach. Finally, we estimated the causal effect of genetically predicted short and long sleep duration on hyperuricemia using genetic variants associated with short and long sleep duration, respectively.

## Materials and methods

### Study participants

The UK Biobank is a large population-based cohort that recruited over 0.5 million participants aged 37–73 years from 22 assessment centers across the UK between 2006 and 2010 (26). At baseline assessment, participants completed a touchscreen questionnaire, a face-to-face interview, and a series of physical and medical measurements. In addition, biological samples were collected for biochemical analyses and genotyping. More details of the UK Biobank have been published previously (26). The UK Biobank study has ethical approval (ref 11/NW/0382) from the North West Multi-Centre Research Ethics Committee (London, UK), and all participants have signed the written informed consent.

**FIGURE 1:**
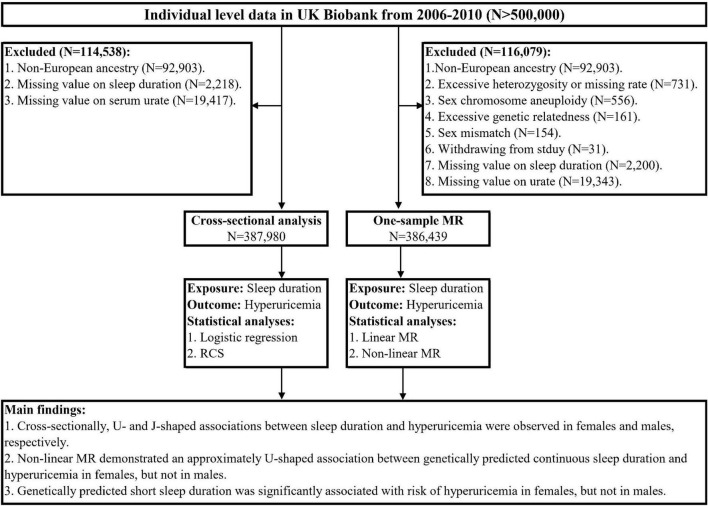
The overall study design. MR, Mendelian randomization; RCS, restricted cubic spline.

The overall study design is presented in Figure 1. For the cross-sectional analysis, we used the baseline data from the UK Biobank. To minimize the population-stratification bias, we restricted the participants to 409,615 individuals of European ancestry as determined by self-report and genetic data. Furthermore, we excluded those with missing values on sleep duration and serum urate at baseline, leaving 387,980 participants in the cross-sectional analysis. For MR analyses, we applied standard quality control for individual-level genetic data to exclude outlier participants. As recommended in a previous study (27), the exclusion criteria included non-white British based on questionnaire and genetic profiling (28), excessive heterozygosity or missing rate, sex chromosome aneuploidy, excessive genetic relatedness, sex mismatch, and withdrawal from the study. We further excluded those with missing values on sleep duration and serum urate, leaving 386,439 participants for subsequent MR analyses.

### Ascertainment of exposure and outcomes

The main exposure of this study was self-reported sleep duration, which was obtained by asking the standardized question: “About how many hours of sleep do you get in every 24 hours? (please include naps),” with the responses expressed in integer hours. The answers of “Do not know” and “Prefer not to answer” would be treated as missing values. To better explore the shape of the association between sleep duration and hyperuricemia in cross-sectional analysis, we categorized sleep duration into five groups: ≤5, 6, 7, 8, ≥9 h/day. Sleep duration of 7 h/day was set as the reference group for subsequent analyses.

This study’s primary outcome was hyperuricemia, defined as serum urate concentrations ≥420 μmol/L in men and ≥360 μmol/L in women (29). Serum urate in participants’ blood samples was measured by the Uricase Pedigree Analysis Package (PAP) on a Beckman Coulter AU5800.

### Covariates

The Townsend deprivation index was used to estimate area deprivation. Education levels were categorized into four groups (college or university, A/AS levels, O/GCSEs levels, and others). Participants’ employment status was classified as employed, retired, and unemployed. Smoking status was grouped into three categories (never, previous, and current). Alcohol consumption was categorized as never or special occasions only, 1–3 times/month, 1–2 times/week, 3–4 times/week, daily, or almost daily. Physical activity was estimated by summed metabolic equivalent task minutes per week (MET-min/week) for all activities, including walking, moderate activity, and vigorous activity. Fruit and vegetable intake (serves/day) was obtained by summing fruit and vegetable intake. High-density lipoprotein cholesterol (HDL-C) was analyzed by enzyme immune-inhibition; low-density lipoprotein cholesterol (LDL-C) was assessed by selective enzymatic protection; total cholesterol (TC) and triglycerides (TGs) were measured by an enzymatic kit; white blood cell (WBC) count was directly measured by the Hematology Analyzer. Obesity was defined as a body mass index ≥30 kg/m^2^ (30). Hypertension was determined by systolic blood pressure ≥140 mmHg, diastolic blood pressure ≥90 mmHg, or self-reported anti-hypertensive drug use (31). Diabetes mellitus was determined by self-report of the physician’s diagnosis.

### Cross-sectional analysis

Logistic regressions were performed to assess the observational association between sleep duration and hyperuricemia in the univariable models. To account for the impact of potential confounders, we conducted multivariable logistic models with adjustments for a number of covariates based on our prior knowledge and previous studies (15–18), including age, sex (only for the model with overall participants), Townsend deprivation index, education level, employment status, smoking status, alcohol consumption, physical activity, fruit and vegetable intake, HDL-C, LDL-C, TG, TC: HDL-C ratio, WBC count, obesity, hypertension, and diabetes mellitus. Odds ratios (ORs) with 95% confidence intervals (95% CIs) and corresponding *P*-values were reported. Furthermore, dose-response associations were evaluated by restricted cubic spline with four knots incorporated into multivariable logistic regression models to visualize the association between sleep duration and hyperuricemia graphically.

All data were expressed as means ± standard deviation (SD) for continuous variables and frequencies (percentages) for categorical variables. Differences in participants’ characteristics across sleep duration categories were assessed using a one-way analysis of variance (ANOVA) for continuous variables or a chi-square test for categorical variables.

### Single-nucleotide polymorphisms and genetic risk score as instrumental variables

A large-scale genome-wide association study (GWAS) in the UK Biobank has identified 78 single-nucleotide polymorphisms (SNPs) associated with continuous sleep duration, 27 SNPs associated with short sleep duration (<7 h *vs*. 7–8 h), and 8 SNPs associated with long sleep duration (>8 h *vs*. 7–8 h) at the GWAS significant level (*P* < 5 × 10^–8^), indicating distinct genetic mechanisms may be involved for the phenotype of short and long sleep duration (32). For further genomic quality control, we excluded SNPs with imputation *r*^2^ (INFO score) <0.9, minor allele frequency <0.01, Hardy-Weinberg *P*-value <1 × 10^–5^, and linkage disequilibrium *r*^2^ > 0.1. As a result, 77 SNPs associated with continuous sleep duration, 26 SNPs associated with short sleep duration, and 7 SNPs associated with long sleep duration were included for subsequent MR analyses (Supplementary Tables 1–3). The unweighted genetic risk score (GRS) was generated as the total number of sleep duration-increasing alleles.

### Linear Mendelian randomization analysis

The two-stage method was used to assess the association between genetically predicted continuous sleep duration and hyperuricemia in the linear MR analysis. Specifically, we first used the linear regression model to estimate the association of the GRS with sleep duration. Then, we regressed hyperuricemia on the predicted values of sleep duration from the first stage using a logistic regression model. Both regression models were adjusted for age, sex (only for the models with overall participants), assessment centers, top 10 genetic principal components, and genotyping array. To account for potential pleiotropy, we used the Mendelian randomization-Egger (MR-Egger) regression intercept to estimate the magnitude of horizontal pleiotropy (33). The significant intercept difference from zero suggests the existence of average horizontal pleiotropy. To avoid the possibility of violating MR assumptions, we performed the Mendelian Randomization Pleiotropy RESidual Sum and Outlier (MR-PRESSO) test to identify the potential outliers (34). Once the outliers were identified, the MR analysis would be re-conducted after the removal of outliers (Supplementary Table 4).

### Non-linear Mendelian randomization analysis

To characterize the shape of the association between genetically predicted continuous sleep duration and hyperuricemia, we employed a piecewise linear MR method (25). Specifically, the sample was first divided into three strata using the instrumental variable-free sleep duration, which was obtained by taking the residuals of sleep duration after regressing on GRS (35). In each stratum, linear MR was performed to estimate the localized average causal effect (LACE) using the ratio of coefficients derived from the associations of GRS with hyperuricemia and sleep duration. Finally, the piecewise linear function was fitted into each stratum, which was constrained to be continuous by taking each line segment to begin where the previous segment finished. The *P*-values from two tests for non-linearity were reported: Cochran’s *Q* test, which assesses the heterogeneity of the LACE estimates, and the quadratic test, which assesses whether a non-linear model fits the LACE estimates better than a linear model (25).

### Genetically predicted short and long sleep duration with the risk of hyperuricemia

Although the non-linear MR could characterize the shape of the causal association between sleep duration and the risk of hyperuricemia, it was not easy to provide the interpretable effect sizes of the causal estimates (36). Therefore, given the potential non-linear association between sleep duration and hyperuricemia, we further included 26 SNPs associated with short sleep duration and seven SNPs associated with long sleep duration to estimate the causal effects of genetically predicted short and long sleep duration on hyperuricemia. The causal estimates were rescaled to be interpreted for each doubling of genetic liability for short and long sleep durations (37). As described in the preceding text, potential horizontal pleiotropy would be detected, and corrected estimates would be obtained by removing outliers (Supplementary Table 4).

To account for multiple testing, we used the Bonferroni correction. Significant results before but not after correction for multiple testing were considered suggestive associations. All statistical analyses were performed with R version 4.1.1 (R Foundation for Statistical Computing, Vienna, Austria) and the “TwoSampleMR,” “MR PRESSO,” and “nlmr” packages.

## Results

### The characteristics of study participants

The characteristics of study participants across categories of sleep duration are presented in Table 1. Compared to those with extreme sleep duration, participants with reference sleep duration (7 h/day) tended to be younger, educated, employed, less deprived, and never smokers. They were also less likely to suffer from obesity, diabetes mellitus, and hypertension.

**TABLE 1:** The characteristics of study participants across categories of sleep duration.

Characteristic	Sleep duration				
	≤5 h/day (N = 19,908)	6 h/day (N = 72,581)	7 h/day (N = 151,393)	8 h/day (N = 114,170)	≥9 h/day (N = 29,928)
**Age, years**	57.3 ± 7.7	56.7 ± 7.8	56.2 ± 8.0	57.4 ± 8.1	58.9 ± 7.8
**Gender, *n* (%)**					
Male	8,687 (43.6%)	3,4642 (47.7%)	72,038 (47.6%)	50,112 (43.9%)	13,147 (43.9%)
Female	11,221 (56.4%)	37,939 (52.3%)	79,355 (52.4%)	64,058 (56.1%)	16,781 (56.1%)
**Education level, *n* (%)**					
College or University degree	3,680 (26.5%)	20,599 (35.3%)	53,316 (41.0%)	35,151 (37.8%)	6,855 (31.7%)
A/AS levels	1,761 (12.7%)	7,914 (13.6%)	18,333 (14.1%)	12,697 (13.7%)	2,841 (13.2%)
O/GCSEs levels	4,423 (31.8%)	16,544 (28.4%)	33,637 (25.9%)	25,248 (27.1%)	6,311 (29.2%)
Others	4,040 (29.1%)	13,272 (22.8%)	24,622 (19.0%)	19,921 (21.4%)	5,597 (25.9%)
**Employment status, *n* (%)**					
Employed	9,936 (50.3%)	44,694 (62.0%)	97,122 (64.6%)	58,013 (51.2%)	9,651 (32.5%)
Retired	6,835 (34.6%)	22,028 (30.6%)	44,998 (29.9%)	46,886 (41.4%)	15,898 (53.6%)
Unemployed	2,971 (15.0%)	5,336 (7.4%)	8,331 (5.5%)	8,359 (7.4%)	4,118 (13.9%)
Townsend deprivation index*	−0.6 ± 3.4	−1.3 ± 3.0	−1.7 ± 2.8	−1.7 ± 2.8	−1.3 ± 3.1
**Smoking status, *n* (%)**					
Never	9,835 (49.7%)	37,987 (52.5%)	84,982 (56.3%)	63,124 (55.5%)	15,103 (50.7%)
Previous	6,915 (34.9%)	25,930 (35.9%)	52,079 (34.5%)	40,375 (35.5%)	11,272 (37.8%)
Current	3,049 (15.4%)	8,389 (11.6%)	13,911 (9.2%)	10,304 (9.1%)	3,437 (11.5%)
**Alcohol consumption, n (%)**					
Never or special occasions only	5,317 (26.8%)	13,004 (17.9%)	22,024 (14.6%)	19,093 (16.7%)	6,827 (22.8%)
1–3 times/month	2,429 (12.2%)	8,461 (11.7%)	16,487 (10.9%)	12,329 (10.8%)	3,292 (11.0%)
1–2 times/week	5,016 (25.2%)	19,346 (26.7%)	40,551 (26.8%)	30,286 (26.5%)	7,371 (24.6%)
3–4 times/week	3,637 (18.3%)	16,761 (23.1%)	39,503 (26.1%)	27,668 (24.2%)	6,023 (20.1%)
Daily or almost daily	3,473 (17.5%)	14,957 (20.6%)	32,764 (21.7%)	24,736 (21.7%)	6,393 (21.4%)
**Physical activity, MET-minutes/week**	2,965.6 ± 3,255.0	2,773.2 ± 2,895.3	2,621.9 ± 2,629.9	2,690.6 ± 2,659.9	2,530.7 ± 2,647.5
**Fruit and vegetable intake, serves/day**	7.8 ± 5.1	7.7 ± 4.6	7.7 ± 4.3	7.8 ± 4.3	7.7 ± 4.5
**WBC count**	7.1 ± 2.0	6.9 ± 2.1	6.8 ± 2.1	6.9 ± 2.0	7.1 ± 2.2
**HDL-C, mmol/L**	1.4 ± 0.4	1.4 ± 0.4	1.5 ± 0.4	1.5 ± 0.4	1.4 ± 0.4
**LDL-C, mmol/L**	3.6 ± 0.9	3.6 ± 0.9	3.6 ± 0.9	3.6 ± 0.9	3.5 ± 0.9
**TGs, mmol/L**	1.9 ± 1.1	1.8 ± 1.0	1.7 ± 1.0	1.8 ± 1.0	1.9 ± 1.1
**TC: HDL-C ratio**	4.2 ± 1.2	4.2 ± 1.1	4.1 ± 1.1	4.1 ± 1.1	4.2 ± 1.2
**Urate, μmol/L**	312.5 ± 82.5	312.2 ± 80.0	307.9 ± 79.3	307.3 ± 80.4	315.2 ± 83.7
**Obesity, n (%)**	6,514 (32.9%)	19,665 (27.2%)	33,061 (21.9%)	25,427 (22.3%)	8,842 (29.7%)
**Diabetes mellitus, n (%)**	1,421 (7.2%)	3,545 (4.9%)	5,876 (3.9%)	5,307 (4.7%)	2,492 (8.4%)
**Hypertension, n (%)**	10,880 (54.7%)	38,503 (53.1%)	77,840 (51.4%)	61,186 (53.6%)	16,942 (56.7%)
**Hyperuricemia, n (%)**	2,976 (14.9%)	9,756 (13.4%)	18,044 (11.9%)	14,604 (12.8%)	4,826 (16.1%)

MET, metabolic equivalent task; WBC, white blood cell; HDL-C, high-density lipoprotein cholesterol; LDL-C, low-density lipoprotein cholesterol; TGs, triglycerides; TC, total cholesterol. Data are presented as means ± standard deviation (SD) for continuous variables and frequencies (percentages) for categorical variables. Participants’ characteristics were compared using one-way ANOVA for continuous variables and the chi-square test for categorical variables. All *P*-value for comparison < 0.001.

*Higher value indicated worse deprivation.

### The observational association of sleep duration with hyperuricemia

**FIGURE 2:**
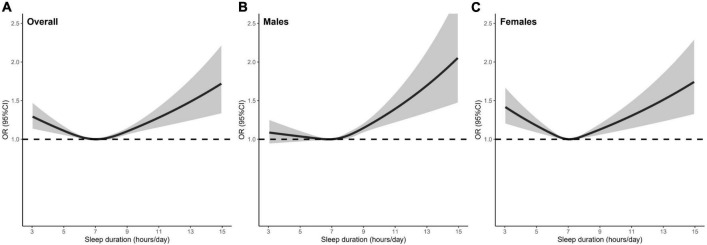
The shape of the association between sleep duration and hyperuricemia in **(A)** overall participants, **(B)** males, and **(C)** females. The dashed line is the reference line at OR = 1. The solid curve and the shaded areas stand for the ORs and their 95% CIs, respectively. ORs were adjusted for age, sex (only for the model with overall participants), Townsend deprivation index, education level, employment status, smoking status, alcohol consumption, physical activity, fruit and vegetable intake, HDL-C, LDL-C, TG, TC: HDL-C ratio, WBC count, obesity, hypertension, diabetes mellitus. OR, odds ratio; HDL-C, high-density lipoprotein cholesterol; LDL-C, low-density lipoprotein cholesterol; TGs, triglycerides; TC, total cholesterol; WBC, white blood cell.

The prevalence of hyperuricemia was 14.1, 17.2, and 9.3% for overall participants, males and females. Table 2 presents the observational association of sleep duration with hyperuricemia. The association was attenuated after adjusting for all potential confounders in the multivariable model. In overall participants and females, both short and long sleep duration consistently had an increased risk for hyperuricemia, even with further adjustment for potential confounders, when compared with the reference group of 7 h/day. The fully adjusted OR [95% CI] of ≤5, ≥9 h/day were 1.11 [1.04–1.19], 1.13 [1.07–1.19] for overall participants, and 1.18 [1.06–1.30], 1.20 [1.10–1.31] for females, respectively. The restricted cubic spline curve analysis revealed the U-shaped associations between sleep duration and hyperuricemia in overall participants and females (Figure 2). In males, although significant associations of both short and long sleep duration with a risk of hyperuricemia were observed in the univariable model, only long sleep duration was significantly associated with an increased risk of hyperuricemia in the multivariable model (OR [95% CI]: 1.16 [1.08–1.24] for ≥9 h/day). The dose-response association between sleep duration and hyperuricemia in males showed an approximately J-shape (Figure 2).

**TABLE 2:** The observational association between sleep duration and hyperuricemia.

Sleep duration	Participants (%)	Univariable model		Multivariable model^§^	
		OR (95% CI)	*P*-value	OR (95% CI)	*P*-value
**Overall**					
≤5 h/day	19,908 (5.1%)	1.30 (1.25–1.35)	<0.001	1.11 (1.04–1.19)	0.001
6 h/day	72,581 (18.7%)	1.15 (1.12–1.18)	<0.001	1.04 (1.01–1.08)	0.024
7 h/day	151,393 (39.0%)	Ref	–	Ref	–
8 h/day	114,170 (29.4%)	1.08 (1.06–1.11)	<0.001	1.03 (1.00–1.07)	0.055
≥9 h/day	29,928 (7.7%)	1.42 (1.37–1.47)	<0.001	1.13 (1.07–1.19)	<0.001
**Males**					
≤5 h/day	8,687 (4.9%)	1.18 (1.12–1.25)	<0.001	1.05 (0.97–1.15)	0.219
6 h/day	34,642 (19.4%)	1.06 (1.03–1.10)	<0.001	0.99 (0.94–1.03)	0.561
7 h/day	72,038 (40.3%)	Ref	–	Ref	–
8 h/day	50,112 (28.1%)	1.09 (1.06–1.13)	<0.001	1.04 (1.00–1.08)	0.051
≥9 h/day	13,147 (7.4%)	1.35 (1.29–1.42)	<0.001	1.16 (1.08–1.24)	<0.001
**Females**					
≤5 h/day	11,221 (5.4%)	1.58 (1.48–1.68)	<0.001	1.18 (1.06–1.30)	0.002
6 h/day	37,939 (18.1%)	1.29 (1.24–1.35)	<0.001	1.14 (1.07–1.22)	<0.001
7 h/day	79,355 (37.9%)	Ref	–	Ref	–
8 h/day	64,058 (30.6%)	1.15 (1.11–1.20)	<0.001	1.09 (1.03–1.15)	0.002
≥9 h/day	16,781 (8.0%)	1.64 (1.56–1.73)	<0.001	1.20 (1.10–1.31)	<0.001

^§^Adjusted for age, sex (only for the model with overall participants), Townsend deprivation index, education level, employment status, smoking status, alcohol consumption, physical activity, fruit and vegetable intake, HDL-C, LDL-C, TG, TC: HDL-C ratio, WBC count, obesity, hypertension, and diabetes mellitus. OR, odds ratio; CI, confidence interval; HDL-C, high-density lipoprotein cholesterol; LDL-C, low-density lipoprotein cholesterol; TGs, triglycerides; TC, total cholesterol; WBC, white blood cell.

### Linear Mendelian randomization analysis of sleep duration with the risk of hyperuricemia

**FIGURE 3:**
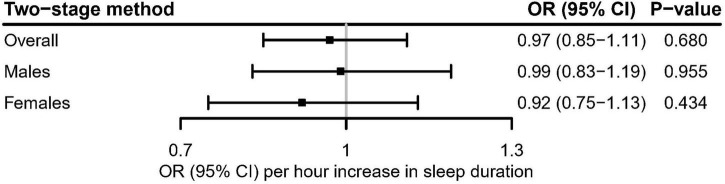
Linear Mendelian randomization estimate for the association between genetically predicted continuous sleep duration and hyperuricemia in the UK Biobank using the two-stage method. After correcting for multiple comparisons, *P* < 0.05/3 = 0.017 was considered significant.

In the linear MR analysis, no evidence was shown for the linear association between genetically predicted continuous sleep duration and the risk of hyperuricemia (Figure 3; OR [95% CI]: 0.97 [0.85–1.11], *P* = 0.680 for overall participants; OR [95% CI]: 0.99 [0.83–1.19], *P* = 0.955 for males; OR [95% CI]: 0.92 [0.75–1.13], *P* = 0.434 for females).

### Non-linear Mendelian randomization analysis of sleep duration with the risk of hyperuricemia

**FIGURE 4:**
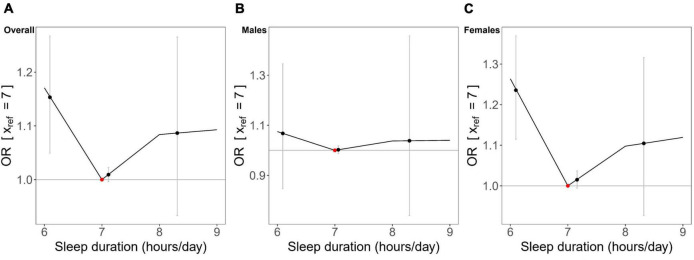
Non-linear Mendelian randomization results between genetically predicted continuous sleep duration and hyperuricemia in **(A)** overall participants, **(B)** males, and **(C)** females. Localized average causal effects (LACE) for hyperuricemia were estimated using the piecewise linear method. Black dots (grey vertical lines) mean the LACE (95% CI) in each stratum; the red dot represents the reference point of sleep duration of 7 hours/day.

In the non-linear MR analysis (Figure 4), we observed approximately U-shaped associations between genetically predicted continuous sleep duration and the risk of hyperuricemia in overall participants (Cochran *Q* test *P* = 0.008; Quadratic test *P* = 0.034) and females (Cochran *Q* test *P* = 0.004; Quadratic test *P* = 0.003), but not in males (Cochran *Q* test *P* = 0.836; Quadratic test *P* = 0.707). The LACE estimates suggested that genetically predicted continuous sleep duration in short sleep duration strata was associated with an increased risk for hyperuricemia in overall participants and females but had not much effect in males (Figure 4). Despite not being statistically significant in the long sleep duration strata of genetically predicted continuous sleep duration, we could still observe the trend for it to increase the risk of hyperuricemia (Figure 4).

### Genetically predicted short and long sleep duration with the risk of hyperuricemia

**FIGURE 5:**
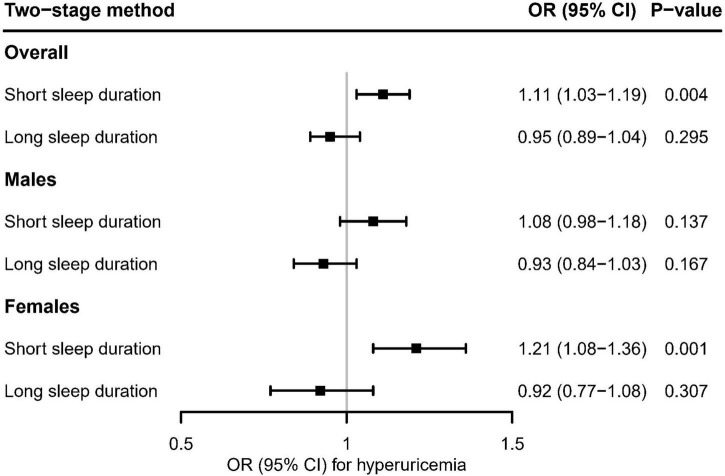
Using the two-stage method, mendelian randomization estimates for genetically predicted short and long sleep duration with hyperuricemia in the UK Biobank. After correcting for multiple comparisons, *P* < 0.05/6 = 0.008 was considered significant.

Figure 5 showed the evidence that genetically predicted short sleep duration had a significant causal adverse effect on the risk of hyperuricemia in overall participants (OR [95% CI]: 1,11 [1.03–1.19], *P* = 0.004) and females (OR [95% CI]: 1.21 [1.08–1.36], *P* = 0.001), but no significant effect in males (OR [95% CI]: 1.08 [0.98–1.18], *P* = 0.137). In contrast, there was no evidence of a significant association between genetically predicted long sleep duration and the risk of hyperuricemia (Figure 5).

### Sensitivity analyses

We performed the following sensitivity analyses to assess the robustness of our findings. First, multivariable generalized additive models were used to examine the observational association between sleep duration and hyperuricemia, as suggested by a reviewer. Similar results were obtained (data not shown). Second, three other MR methods with different assumptions of horizontal pleiotropy were conducted as sensitivity analyses: inverse-variance weighted (IVW) (38), weighted median (WM) (39), and MR-Egger (33). In general, the causal estimates obtained from different MR methods showed consistent direction with those of the main analyses, and the MR-Egger regression intercept did not detect any significant horizontal pleiotropy (Supplementary Tables 5–7 and Supplementary Figures 1–3). Third, we further examined the causal effect of sleep duration on serum urate concentrations (μmol/L) in the UK Biobank. The linear MR analyses could not provide evidence of a significant association between genetically predicted continuous sleep duration and serum urate concentrations (Supplementary Table 8). However, genetically predicted short sleep duration was significantly associated with higher serum urate concentrations in overall participants (β [95% CI]: 2.91 [1.31–4.51]; *P* < 0.001) and females (β [95% CI]: 4.14 [2.03–6.24]; *P* < 0.001), and had nominal effect on urate concentrations in males (β [95% CI]: 2.84 [0.29–5.38]; *P* = 0.029) (Supplementary Table 9). On the contrary, there was no significant association between genetically predicted long sleep duration and serum urate concentrations (Supplementary Table 10). In summary, those results enhanced the robustness of our primary findings that genetically predicted short sleep duration had an adverse causal effect on the risk for hyperuricemia in females but had little effect in males.

## Discussion

Sleep duration was recently considered as a potentially modifiable risk factor for hyperuricemia. To the best of our knowledge, this is the first study that explored the causal association between sleep duration and hyperuricemia using Mendelian randomization. There were three main findings. First, the cross-sectional analysis demonstrated U- and J-shaped associations between sleep duration and hyperuricemia in females and males, respectively. Second, non-linear MR supported an approximately U-shaped association between genetically predicted continuous sleep duration and hyperuricemia in females but not in males. Third, genetically predicted short sleep duration was significantly associated with a risk for hyperuricemia in females but not in males. In contrast, genetically predicted long sleep duration was not adversely associated with the risk of hyperuricemia in both males and females. These findings suggest that short sleep duration may be a modifiable causal risk factor for hyperuricemia in females but has little effect in males. Long sleep duration does not appear to be causally associated with hyperuricemia. Our traditional observational analysis results were consistent with and extended previous work. Several recent observational studies have investigated the associations between sleep duration and hyperuricemia (15–18). Most of these studies supported short sleep duration as a potential risk factor for hyperuricemia (15–17). For example, a study of 4,555 Chinese adults found that short sleep duration was associated with higher urate levels (15). However, only one of these studies explored the association between sleep duration and hyperuricemia in both males and females and found a sex-specific association (16).

Furthermore, another study on Korean women revealed a significant U-shaped association between sleep duration and hyperuricemia, suggesting a potential sex-specific non-linear association (18). Our study’s observational findings supported the significant associations between short and long sleep duration and an increased risk for hyperuricemia in the overall population. The restricted cubic spline plot shows that our observational findings also support the sex-specific non-linear associations between sleep duration and hyperuricemia, which were further confirmed by non-linear MR analyses.

The adverse effect of short sleep duration on hyperuricemia in females was supported by both cross-sectional and MR analyses. However, the exact mechanisms underlying the association between short sleep duration and the risk of hyperuricemia are poorly understood. Several potential explanations may account for this result. First, sleep loss has been demonstrated to be associated with elevated levels of catecholamine (40), which could increase nucleotide turnover and then enhance the production of endogenous urate (41). The potential role of catecholamine in the pathogenesis of hyperuricemia was also illustrated in animal models (42, 43). Second, the proteolytic pathway activated by sleep deprivation could also result in the overproduction of purines and urates (44, 45). Third, previous literature has demonstrated that sleep curtailment may be associated with pathophysiological changes in endocrine, autonomic, and immune systems (40, 46, 47). Those changes could further contribute to an increased risk of several metabolic disorders (e.g., obesity, hypertension) (48–50), which have been considered risk factors for hyperuricemia (51). Although we adjusted for WBC count, TC: HDL-C ratio, obesity, and other metabolic factors in the multivariable model of cross-sectional analysis, we still observed a significant association between short sleep duration and hyperuricemia. Further studies are required to clarify the exact underlying mechanisms of those associations.

The present study demonstrated that the impact of short sleep duration was significant only in females but not in males. Nonetheless, there was no exact evidence of the mechanisms underlying the sex differences. It is generally known that males tend to have higher urate concentrations and a prevalence of hyperuricemia than females (3, 52), which could also be observed in our study. This could partly be explained by sex differences in the distribution of potential risk factors for hyperuricemia. For example, the prevalence of hypertension in males was significantly higher than in females. After adjusting for those potential risk factors, short sleep duration was no longer significantly associated with hyperuricemia in males, which suggested that the high prevalence of hyperuricemia in males might not be caused by short sleep duration. Another explanation may be the impact of dramatic changes in hormonal and metabolic profiles in females after menopause, which have been demonstrated to be associated with a roughly 4-fold increased risk of development of hyperuricemia (52). In the present study, the average age of females was 56.7 years, suggesting that most females in our study were at high risk of developing hyperuricemia. Therefore, those females were more likely to suffer from adverse metabolic outcomes caused by short sleep duration. Further studies are warranted to elucidate whether there were sex-specific associations in younger populations.

Although long sleep duration was observed to be strongly associated with an increased risk for hyperuricemia in our cross-sectional analyses, its causal effects on hyperuricemia risk require further investigation. In the non-linear MR, we could still observe a trend of an increased risk for hyperuricemia in the long sleep duration strata of genetically predicted continuous sleep duration, although it was not statistically significant. However, genetically predicted long sleep duration (>8 h/day *vs*. 7–8 h/day) was not significantly associated with hyperuricemia. Two explanations might account for the discrepancy. First, MR analyses require large-scale samples to estimate causal effects. However, our study’s relatively small number of long sleepers (around 29,000 participants) might lead to relatively low statistical power, raising the concern of false negatives. Second, the limited number of SNPs associated with long sleep duration might give rise to weak instrumental variables. However, the relatively high *F*-statistic for long sleep duration (i.e., *F*-statistic > 10) indicated that the bias induced by weak instrumental variables would be limited. In our study, the estimated *F*-statistics for long sleep duration were 302.26, 180.41, and 124.90 for overall participants, males, and females, respectively. Additionally, the consistency of the results in different MR methods confirmed the limited bias effect. Therefore, we recommend further large-scale studies on the underlying mechanisms between long sleep duration and hyperuricemia or urate concentrations rather than reducing sleep duration in a population with long sleeping behavior to prevent hyperuricemia.

Our study has certain strengths. First, to our knowledge, this is the first study to detect the causality between sleep duration and hyperuricemia. The MR design could effectively minimize the potential biases caused by confounding and reverse causality in the observational study. Second, we applied a state-of-the-art non-linear MR approach to visualize the shape of the causal association between sleep duration and the risk of hyperuricemia, which could overcome the limitation of linear assumptions in the standard MR approach. Furthermore, to provide the interpretable effect sizes of causal estimators, genetic variants associated with short and long sleep duration were used to estimate the causal effect of genetically predicted short and long sleep duration on the risk of hyperuricemia.

Several potential limitations in our study are worth mentioning. First, sleep duration was self-reported by participants, which might result in misclassification due to recall bias. However, obtaining objective measurements, such as using polysomnography, is not feasible in a large-scale cohort study. Moreover, previous studies have demonstrated a moderate relationship between self-reported and actigraphy-measured sleep duration (53, 54). Second, our study did not include other sleep-related characteristics (e.g., sleep apnea and restless legs syndrome), which were potential risk factors for metabolic disorders (55, 56). Also, different sleep characteristics might affect each other (57). Therefore, further studies are required to investigate the causal associations of more characteristics with hyperuricemia or other metabolic disorders. Third, although we excluded potential outliers identified by MR-PRESSO, we cannot completely rule out the possible impact of the horizontal pleiotropic effect on results. However, the MR-Egger intercept test was not statistically significant, indicating that the bias caused by horizontal pleiotropy would be limited. Fourth, the analyses in our study restricted the participants to individuals aged 37–73 years of European ancestry. Although it could minimize the population stratification bias, the generalizability of our findings to other populations should be interpreted with caution. Finally, our study did not provide direct insight into the mechanisms of the adverse effect of short sleep duration on hyperuricemia.

## Conclusion

In conclusion, short sleep duration is a potentially causal risk factor for hyperuricemia for females but does not affect males much. Although long sleep duration is observationally associated with the risk of hyperuricemia, it does not appear to be causally associated with the risk of hyperuricemia. Based on our findings, we suggest that prevention and intervention strategies targeting sleep-deprived individuals to prolong sleep duration may help reduce the risk of hyperuricemia, especially for females. Reducing sleep duration is unlikely to be effective in hyperuricemic prevention and intervention for individuals with long sleep duration. Further study is warranted to generalize our findings to other populations and clarify the exact underlying mechanisms.

## Data availability statement

The original contributions presented in this study are included in the article/Supplementary material, further inquiries can be directed to the corresponding authors.

## Ethics statement

The studies involving human participants were reviewed and approved by the National Health Service’s National Research Ethics Service (ref. 11/NW/0382). The patients/participants provided their written informed consent to participate in this study.

## Author contributions

CZ carried out the literature review and conceptualized the study. CZ, ZW, JL, and WH contributed to the data curation. ZW helped with validation and performed sensitivity analyses. CZ, ZW, VG, YZ, SZ, and GJ interpreted the results and wrote the original draft of the manuscript. ZW, JL, WH, VG, YZ, SZ, JY, LH, and GJ helped review and edit the final draft of the manuscript. CZ, ZW, JL, WH, and GJ verified the underlying data. GJ, LH, and JY are responsible for the decision to submit the manuscript. All authors contributed to the development of the methodology, had full access to all the data in the study, and accepted the responsibility to submit it for publication.
