# Supplementary material for: Linear and non-linear Mendelian randomization analyses of sex-specific associations between sleep duration and hyperuricemia
Source: Front Nutr. 2022 Oct 10;9:920791. doi: 10.3389/fnut.2022.920791 (PMC9632753; doi:10.3389/fnut.2022.920791)
Supplement: Supplementary file 1 [file Data_Sheet_1.docx]

***Supplementary Materials***

**Linear and Non-linear Mendelian Randomization Analyses of Sex-specific Association Between Sleep Duration and Hyperuricemia**

**Table of Contents**

[Table S1. Genetic information on 77 SNPs associated with continuous sleep duration in UK Biobank. 2](#_Toc112486264)

[Table S2. Genetic information on 26 SNPs associated with short sleep duration in UK Biobank. 4](#_Toc112486265)

[Table S3. Genetic information on 7 SNPs associated with long sleep duration in UK Biobank. 5](#_Toc112486266)

[Table S5. Associations of genetically predicted continuous sleep duration with risk of hyperuricemia using IVW, MR-Egger, and WM methods. 7](#_Toc112486267)

[Table S6. Associations of genetically predicted short sleep duration with risk of hyperuricemia using IVW, MR-Egger, and WM methods. 8](#_Toc112486268)

[Table S7. Associations of genetically predicted long sleep duration with risk of hyperuricemia using IVW, MR-Egger, and WM methods. 9](#_Toc112486269)

[Table S8. Association of genetically predicted continuous sleep duration with serum urate concentration (µmol/L) using two-stage, IVW, MR-Egger, and WM methods. 10](#_Toc112486270)

[Table S9. Association of genetically predicted short sleep duration with serum urate concentrations (µmol/L) using two-stage, IVW, MR-Egger, and WM methods. 11](#_Toc112486271)

[Table S10. Association of genetically predicted long sleep duration with serum urate concentration (µmol/L) using two-stage, IVW, MR-Egger, and WM methods. 12](#_Toc112486272)

[Figure S1. Scatter plot of association estimates for genetically predicted sleep duration and risk of hyperuricemia in overall participants, males, and females. 13](#_Toc112486273)

[Figure S2. Scatter plot of association estimates for genetically predicted short sleep duration and risk of hyperuricemia. 14](#_Toc112486274)

[Figure S3. Scatter plot of association estimates for genetically predicted long sleep duration and risk of hyperuricemia. 15](#_Toc112486275)

Table S1. Genetic information on 77 SNPs associated with continuous sleep duration in UK Biobank.

| SNP | CHR | POS | EA | OA | EAF | Beta | SE | *P*-value |
| --- | --- | --- | --- | --- | --- | --- | --- | --- |
| rs12567114 | 1 | 98527951 | A | G | 0.275802 | 0.014831 | 0.00254 | 4.30E-09 |
| rs269054 | 1 | 57864304 | A | T | 0.422076 | 0.013643 | 0.002293 | 2.10E-09 |
| rs61796569 | 1 | 66476437 | T | C | 0.269583 | 0.015444 | 0.002564 | 1.50E-09 |
| rs915416 | 1 | 34731984 | C | G | 0.289947 | 0.019259 | 0.002495 | 9.90E-15 |
| rs10173260 | 2 | 210377845 | C | T | 0.606235 | 0.012837 | 0.002313 | 2.90E-08 |
| rs11885663 | 2 | 166944004 | T | C | 0.247809 | 0.016218 | 0.002618 | 8.60E-10 |
| rs12611523 | 2 | 139195328 | A | G | 0.545244 | 0.012635 | 0.002276 | 3.10E-08 |
| rs374153 | 2 | 40382712 | C | T | 0.158085 | 0.017612 | 0.003103 | 9.10E-09 |
| rs4128364 | 2 | 147612734 | C | T | 0.339025 | 0.014595 | 0.002391 | 1.40E-09 |
| rs4538155 | 2 | 157040773 | T | C | 0.647426 | 0.012975 | 0.002374 | 3.60E-08 |
| rs62120041 | 2 | 9185564 | T | C | 0.933902 | 0.026111 | 0.004575 | 9.60E-09 |
| rs72804080 | 2 | 59358659 | G | A | 0.149928 | 0.017794 | 0.003196 | 2.90E-08 |
| rs75539574 | 2 | 58871658 | C | A | 0.085792 | 0.036248 | 0.004065 | 6.90E-19 |
| rs7556815 | 2 | 114085785 | A | G | 0.219144 | 0.040725 | 0.00274 | 1.30E-49 |
| rs112230981 | 3 | 55879269 | A | G | 0.94984 | 0.031528 | 0.005228 | 2.20E-09 |
| rs13088093 | 3 | 135838598 | G | T | 0.336317 | 0.016272 | 0.002402 | 7.00E-12 |
| rs17732997 | 3 | 70470834 | C | G | 0.569098 | 0.012935 | 0.002288 | 1.20E-08 |
| rs7616632 | 3 | 137031237 | T | G | 0.522135 | 0.013204 | 0.002269 | 4.30E-09 |
| rs7644809 | 3 | 107564459 | T | C | 0.421606 | 0.013062 | 0.002301 | 1.60E-08 |
| rs13109404 | 4 | 102896591 | T | G | 0.928024 | 0.031204 | 0.004408 | 1.40E-12 |
| rs17427571 | 4 | 82254908 | A | G | 0.684313 | 0.013826 | 0.002435 | 1.30E-08 |
| rs2192528 | 4 | 18327896 | A | G | 0.480065 | 0.013369 | 0.002269 | 2.70E-09 |
| rs35531607 | 4 | 92533225 | C | T | 0.474083 | 0.01284 | 0.002273 | 1.50E-08 |
| rs11567976 | 5 | 137654218 | T | C | 0.570908 | 0.012804 | 0.002285 | 2.10E-08 |
| rs151014368 | 5 | 176751059 | A | G | 0.206258 | 0.016092 | 0.00282 | 9.10E-09 |
| rs180769 | 5 | 135615615 | T | C | 0.424698 | 0.012724 | 0.002294 | 2.30E-08 |
| rs365663 | 5 | 1428883 | A | G | 0.545963 | 0.014629 | 0.002279 | 1.00E-10 |
| rs460692 | 5 | 3126584 | C | T | 0.137484 | 0.021056 | 0.003332 | 3.60E-10 |
| rs56372231 | 5 | 102321905 | T | C | 0.334093 | 0.016944 | 0.0024 | 2.20E-12 |
| rs113113059 | 6 | 43160375 | T | C | 0.78 | 0.016141 | 0.002737 | 8.40E-09 |
| rs2231265 | 6 | 89790201 | G | A | 0.772289 | 0.014955 | 0.002699 | 2.70E-08 |
| rs34556183 | 6 | 28584775 | A | G | 0.719606 | 0.016923 | 0.002523 | 2.30E-11 |
| rs80193650 | 6 | 33464363 | G | A | 0.162466 | 0.016839 | 0.003067 | 4.10E-08 |
| rs9345234 | 6 | 93162639 | C | A | 0.578016 | 0.013012 | 0.002299 | 1.80E-08 |
| rs9382445 | 6 | 54937974 | T | C | 0.62305 | 0.014536 | 0.002334 | 4.80E-10 |
| rs2079070 | 7 | 114126432 | C | G | 0.264613 | 0.017548 | 0.002566 | 7.50E-12 |
| rs34731055 | 7 | 2106928 | T | C | 0.18089 | 0.01946 | 0.002948 | 3.70E-11 |
| rs7806045 | 7 | 132610266 | T | C | 0.754703 | 0.014792 | 0.002626 | 1.40E-08 |
| rs330088 | 8 | 9149746 | C | T | 0.547012 | 0.014469 | 0.002277 | 2.70E-10 |
| rs73219758 | 8 | 14279446 | G | A | 0.708064 | 0.016401 | 0.002495 | 5.60E-11 |
| rs10973207 | 9 | 37100525 | T | G | 0.157677 | 0.020434 | 0.003124 | 6.00E-11 |
| rs1776776 | 9 | 140497072 | T | C | 0.873832 | 0.019963 | 0.003411 | 4.90E-09 |
| rs10761674 | 10 | 64618340 | C | T | 0.477334 | 0.012333 | 0.002266 | 4.20E-08 |
| rs11190970 | 10 | 103128332 | G | A | 0.798661 | 0.015379 | 0.002823 | 4.60E-08 |
| rs12246842 | 10 | 21830580 | A | G | 0.459815 | 0.013395 | 0.002274 | 3.90E-09 |
| rs7915425 | 10 | 125016501 | T | C | 0.174682 | 0.019064 | 0.00299 | 2.00E-10 |
| rs1057703 | 11 | 122830251 | G | T | 0.146608 | 0.019402 | 0.003207 | 1.10E-09 |
| rs11602180 | 11 | 48162453 | C | T | 0.836621 | 0.018242 | 0.003066 | 2.30E-09 |
| rs1263056 | 11 | 116576415 | A | G | 0.519099 | 0.012799 | 0.002277 | 2.00E-08 |
| rs12791153 | 11 | 80685181 | T | A | 0.081089 | 0.023548 | 0.004217 | 1.90E-08 |
| rs1517572 | 11 | 28829882 | C | A | 0.580536 | 0.014644 | 0.002295 | 1.50E-10 |
| rs1553132 | 11 | 88297740 | G | A | 0.258433 | 0.014507 | 0.002584 | 2.50E-08 |
| rs174560 | 11 | 61581764 | C | T | 0.314215 | 0.013575 | 0.002437 | 2.80E-08 |
| rs1939455 | 11 | 101520886 | G | T | 0.879446 | 0.020425 | 0.003561 | 1.20E-08 |
| rs4592416 | 11 | 43800474 | G | A | 0.464407 | 0.014683 | 0.00227 | 9.30E-11 |
| rs7115226 | 11 | 113408518 | A | C | 0.073525 | 0.026573 | 0.004358 | 1.70E-09 |
| rs7951019 | 11 | 118358027 | G | T | 0.032227 | 0.036879 | 0.006521 | 1.20E-08 |
| rs11614986 | 12 | 110007939 | A | G | 0.820952 | 0.016379 | 0.002951 | 2.70E-08 |
| rs34354917 | 12 | 38764559 | C | A | 0.710472 | 0.013746 | 0.002501 | 3.90E-08 |
| rs4767550 | 12 | 117951150 | G | A | 0.414138 | 0.0143 | 0.00231 | 6.30E-10 |
| rs10483350 | 14 | 29816155 | G | A | 0.195418 | 0.017369 | 0.002868 | 1.50E-09 |
| rs11621908 | 14 | 78495761 | C | T | 0.917141 | 0.024095 | 0.004163 | 5.60E-09 |
| rs55658675 | 14 | 65554638 | C | T | 0.644938 | 0.013142 | 0.002369 | 2.00E-08 |
| rs61985058 | 14 | 60233841 | T | C | 0.143176 | 0.018594 | 0.003229 | 1.30E-08 |
| rs6575005 | 14 | 26954078 | T | C | 0.757854 | 0.015564 | 0.002642 | 4.40E-09 |
| rs8038326 | 15 | 47989799 | A | G | 0.72691 | 0.01592 | 0.002541 | 2.80E-10 |
| rs11643715 | 16 | 23909538 | G | C | 0.290942 | 0.013895 | 0.002497 | 3.20E-08 |
| rs3095508 | 16 | 6550400 | C | A | 0.593529 | 0.015352 | 0.002304 | 3.10E-11 |
| rs8050478 | 16 | 56120461 | G | A | 0.500253 | 0.016001 | 0.002265 | 1.70E-12 |
| rs9940646 | 16 | 53800629 | C | G | 0.577569 | 0.016946 | 0.002291 | 1.20E-13 |
| rs1991556 | 17 | 44083402 | G | A | 0.773765 | 0.016566 | 0.002724 | 1.00E-09 |
| rs205024 | 17 | 11227352 | T | C | 0.383735 | 0.013826 | 0.002327 | 3.90E-09 |
| rs7503199 | 17 | 8134275 | C | T | 0.734267 | 0.014745 | 0.002564 | 1.00E-08 |
| rs9903973 | 17 | 50571227 | C | T | 0.46702 | 0.012775 | 0.002272 | 2.60E-08 |
| rs12607679 | 18 | 53059748 | T | C | 0.737717 | 0.020139 | 0.002593 | 8.30E-15 |
| rs10421649 | 19 | 9942262 | A | T | 0.55697 | 0.013298 | 0.002295 | 6.90E-09 |
| rs2072727 | 20 | 43538733 | T | C | 0.43617 | 0.013243 | 0.002285 | 7.90E-09 |

Abbreviations: SNP, single-nucleotide polymorphism; CHR, chromosome; POS, position; EA, effect allele; OA, other allele; EAF, effect allele frequency; SE, standard error.

Table S2. Genetic information on 26 SNPs associated with short sleep duration in UK Biobank.

| SNP | CHR | POS | EA | OA | EAF | Beta | SE | *P*-value |
| --- | --- | --- | --- | --- | --- | --- | --- | --- |
| rs12567114 | 1 | 98527951 | G | A | 0.7246 | 0.006325 | 0.001077 | 4.10E-09 |
| rs2186122 | 1 | 66470206 | T | A | 0.561566 | 0.00567 | 0.000972 | 4.80E-09 |
| rs2820313 | 1 | 201870221 | G | A | 0.341112 | 0.006006 | 0.00101 | 2.30E-09 |
| rs7524118 | 1 | 34736052 | C | T | 0.708376 | 0.005762 | 0.001054 | 4.90E-08 |
| rs1380703 | 2 | 57941287 | G | A | 0.383531 | 0.006764 | 0.001005 | 1.60E-11 |
| rs2863957 | 2 | 114089551 | C | A | 0.781508 | 0.01019 | 0.001161 | 2.60E-18 |
| rs75539574 | 2 | 58871658 | A | C | 0.914664 | 0.011157 | 0.001727 | 8.40E-11 |
| rs2014830 | 3 | 50172397 | C | T | 0.698128 | 0.005786 | 0.00105 | 2.70E-08 |
| rs13107325 | 4 | 103188709 | T | C | 0.074528 | 0.013268 | 0.001828 | 2.50E-13 |
| rs17005118 | 4 | 82288564 | A | G | 0.264936 | 0.006482 | 0.001087 | 2.50E-09 |
| rs12518468 | 5 | 7249696 | C | T | 0.328456 | 0.005885 | 0.001021 | 8.50E-09 |
| rs3776864 | 5 | 102327868 | A | C | 0.66721 | 0.005724 | 0.001019 | 1.70E-08 |
| rs4585442 | 5 | 135508381 | G | A | 0.311023 | 0.006347 | 0.001036 | 8.10E-10 |
| rs12661667 | 6 | 41792545 | T | C | 0.263495 | 0.006022 | 0.001087 | 2.80E-08 |
| rs9321171 | 6 | 129848635 | C | T | 0.540122 | 0.005354 | 0.000966 | 4.20E-08 |
| rs9367621 | 6 | 55040290 | T | A | 0.43104 | 0.005445 | 0.00097 | 1.60E-08 |
| rs11763750 | 7 | 2080114 | G | A | 0.814346 | 0.007212 | 0.001234 | 5.10E-09 |
| rs1229762 | 7 | 114218582 | T | C | 0.664501 | 0.007239 | 0.001017 | 1.00E-12 |
| rs60882754 | 8 | 52886619 | A | T | 0.938985 | 0.011304 | 0.002001 | 1.80E-08 |
| rs1607227 | 11 | 28808617 | G | T | 0.704938 | 0.006369 | 0.001055 | 1.50E-09 |
| rs7939345 | 11 | 47980568 | T | G | 0.207569 | 0.006498 | 0.001182 | 4.00E-08 |
| rs17388803 | 15 | 48027204 | C | A | 0.105648 | 0.009826 | 0.001587 | 6.50E-10 |
| rs59779556 | 16 | 56227965 | T | G | 0.553827 | 0.005491 | 0.000966 | 2.00E-08 |
| rs205024 | 17 | 11227352 | C | T | 0.616724 | 0.00551 | 0.000986 | 2.70E-08 |
| rs12963463 | 18 | 53099093 | C | T | 0.299425 | 0.007114 | 0.00106 | 1.90E-11 |
| rs5757675 | 22 | 39838892 | G | T | 0.259528 | 0.006455 | 0.001099 | 2.70E-09 |

Abbreviation: SNP, single-nucleotide polymorphisms; CHR, chromosome; POS, position; EA, effect allele; OA, other allele; EAF, effect allele frequency; SE, standard allele.

Table S3. Genetic information on 7 SNPs associated with long sleep duration in UK Biobank.

| SNP | CHR | POS | EA | OA | EAF | Beta | SE | *P*-value |
| --- | --- | --- | --- | --- | --- | --- | --- | --- |
| rs7534398 | 1 | 7767464 | A | T | 0.201382 | 0.005079 | 0.000908 | 2.10E-08 |
| rs6737318 | 2 | 114083120 | G | A | 0.221841 | 0.00638 | 0.000877 | 3.40E-13 |
| rs10899257 | 11 | 76415209 | A | G | 0.144473 | 0.005638 | 0.001031 | 4.60E-08 |
| rs3751046 | 11 | 122828342 | G | A | 0.147342 | 0.005775 | 0.001027 | 2.00E-08 |
| rs75458655 | 11 | 118115331 | T | C | 0.022973 | 0.016731 | 0.002423 | 5.40E-12 |
| rs17817288 | 16 | 53807764 | A | G | 0.518127 | 0.004188 | 0.000727 | 8.90E-09 |
| rs17688916 | 17 | 43778680 | T | A | 0.796267 | 0.006209 | 0.000917 | 1.10E-11 |

Abbreviation: SNP, single-nucleotide polymorphisms; CHR, chromosome; POS, position; EA, effect allele; OA, other allele; EAF, effect allele frequency; SE, standard allele.

**Table S4. The potential outliers identified by MR-PRESSO.**

| **Exposure** | **Population** | **The potential outliers** |
| --- | --- | --- |
| Continuous sleep duration | Overall | rs34556183, rs174560, rs9940646 |
|  | Males | \ |
|  | Females | rs34556183, rs9940646, rs7503199 |
|  |  |  |
| Short sleep duration | Overall | rs13107325, rs2014830 |
|  | Males | rs13107325, rs17005118, rs2014830 |
|  | Females | rs13107325, rs17388803, rs5757675 |
|  |  |  |
| Long sleep duration | Overall | rs17817288 |
|  | Males | \ |
|  | Females | rs75458655, rs17817288 |

These potential outliers were identified by MR-PRESSO, and excluded from our MR analyses.

Abbreviation: MR, Mendelian randomization; MR-PRESSO, Mendelian randomization Pleiotropy RESidual Sum and Outliers.

Table S5. Associations of genetically predicted continuous sleep duration with risk of hyperuricemia using IVW, MR-Egger, and WM methods.

| **Population and method** | **OR (95% CI)** | ***P*-value** |
| --- | --- | --- |
| **Overall** |  |  |
| IVW | 0.97 (0.82 - 1.15) | 0.737 |
| MR-Egger | 0.96 (0.56 - 1.67) | 0.897 |
| WM | 0.97 (0.79 - 1.19) | 0.770 |
| MR-Egger intercept* | \ | 0.979 |
| **Males** |  |  |
| IVW | 0.97 (0.79 - 1.19) | 0.756 |
| MR-Egger | 0.79 (0.43 - 1.45) | 0.447 |
| WM | 0.90 (0.69 - 1.17) | 0.439 |
| MR-Egger intercept* | \ | 0.486 |
|  |  |  |
| **Females** |  |  |
| IVW | 0.95 (0.74 - 1.23) | 0.698 |
| MR-Egger | 1.18 (0.58 - 2.43) | 0.649 |
| WM | 0.99 (0.71 - 1.36) | 0.927 |
| MR-Egger intercept* | \ | 0.528 |

Adjusted for age, sex (only for the model with overall participants), assessment centers, top 10 genetic principal components, and genotyping array.

Abbreviations: IVW, inverse-variance weighted; MR-Egger, Mendelian Randomization-Egger; WM, weighted median.

* MR-Egger intercept was used to detect the horizontal pleiotropy. *P*-value <0.05 indicated potential pleiotropy, which suggested exposure-associated genetic variants may influence the outcome through other pathways rather than through exposure.

After correcting for multiple comparison, *P*<0.05/3=0.017 was considered as significant.

Table S6. Associations of genetically predicted short sleep duration with risk of hyperuricemia using IVW, MR-Egger, and WM methods.

| **Population and method** | **OR (95% CI)** | ***P*-value** |
| --- | --- | --- |
| **Overall** |  |  |
| IVW | 1.09 (0.98 - 1.22) | 0.098 |
| MR-Egger | 0.74 (0.43 - 1.26) | 0.279 |
| WM | 1.03 (0.92 - 1.15) | 0.649 |
| MR-Egger intercept* | \ | 0.157 |
|  |  |  |
| **Males** |  |  |
| IVW | 1.07 (0.94 - 1.21) | 0.300 |
| MR-Egger | 0.94 (0.54 - 1.62) | 0.824 |
| WM | 1.10 (0.95 - 1.27) | 0.209 |
| MR-Egger intercept* | \ | 0.640 |
|  |  |  |
| **Females** |  |  |
| IVW | 1.18 (1.00 - 1.39) | 0.045 |
| MR-Egger | 0.71 (0.35 - 1.45) | 0.359 |
| WM | 1.14 (0.95 - 1.37) | 0.155 |
| MR-Egger intercept* | \ | 0.168 |

Adjusted for age, sex (only for the model with overall participants), assessment centers, top 10 genetic principal components, and genotyping array.

Abbreviations: IVW, inverse-variance weighted; MR-Egger, Mendelian Randomization-Egger; WM, weighted median.

* MR-Egger intercept was used to detect the horizontal pleiotropy. *P*-value <0.05 indicated potential pleiotropy, which suggested exposure-associated genetic variants may influence the outcome through other pathways rather than through exposure.

After correcting for multiple comparison, *P*<0.05/3=0.017 was considered as significant.

Table S7. Associations of genetically predicted long sleep duration with risk of hyperuricemia using IVW, MR-Egger, and WM methods.

| **Population and method** | **OR (95% CI)** | ***P*-value** |
| --- | --- | --- |
| **Overall** |  |  |
| IVW | 0.95 (0.87 - 1.04) | 0.283 |
| MR-Egger | 0.94 (0.66 - 1.32) | 0.726 |
| WM | 0.93 (0.83 - 1.05) | 0.230 |
| MR-Egger intercept* | \ | 0.931 |
|  |  |  |
| **Males** |  |  |
| IVW | 0.93 (0.80 - 1.07) | 0.304 |
| MR-Egger | 0.90 (0.56 - 1.44) | 0.674 |
| WM | 0.85 (0.73 - 0.99) | 0.032 |
| MR-Egger intercept* | \ | 0.896 |
|  |  |  |
| **Females** |  |  |
| IVW | 0.93 (0.79 - 1.09) | 0.369 |
| MR-Egger | 1.36 (0.51 - 3.60) | 0.583 |
| WM | 0.98 (0.79 - 1.23) | 0.878 |
| MR-Egger intercept* | \ | 0.493 |

Adjusted for age, sex (only for the model with overall participants), assessment centers, top 10 genetic principal components, and genotyping array.

Abbreviations: IVW, inverse-variance weighted; MR-Egger, Mendelian Randomization-Egger; WM, weighted median.

* MR-Egger intercept was used to detect the horizontal pleiotropy. *P*-value <0.05 indicated potential pleiotropy, which suggested exposure-associated genetic variants may influence the outcome through other pathways rather than through exposure.

After correcting for multiple comparison, *P*<0.05/3=0.017 was considered as significant.

Table S8. Association of genetically predicted continuous sleep duration with serum urate concentration (µmol/L) using two-stage, IVW, MR-Egger, and WM methods.

| **Population and method** | **β (95% CI)** | ***P*-value** |
| --- | --- | --- |
| **Overall** |  |  |
| Two-stage | 1.97 (-1.10 - 5.04) | 0.209 |
| IVW | 1.58 (-2.98 - 6.14) | 0.497 |
| MR-Egger | -2.08 (-16.95 - 12.79) | 0.785 |
| WM | 1.69 (-3.10 - 6.48) | 0.490 |
| MR-Egger intercept* | \ | 0.612 |
|  |  |  |
| **Males** |  |  |
| Two-stage | 2.72 (-2.20 - 7.63) | 0.278 |
| IVW | 2.55 (-3.45 - 8.57) | 0.404 |
| MR-Egger | 1.40 (-16.09 - 18.89) | 0.876 |
| WM | -0.71 (-7.86 - 6.44) | 0.846 |
| MR-Egger intercept* | \ | 0.890 |
|  |  |  |
| **Females** |  |  |
| Two-stage | 1.73 (-2.06 - 5.52) | 0.372 |
| IVW | 0.50 (-4.98 - 5.99) | 0.857 |
| MR-Egger | -8.07 (-23.56 - 7.42) | 0.311 |
| WM | -2.42 (-8.31 - 3.47) | 0.421 |
| MR-Egger intercept* | \ | 0.250 |

Adjusted for age, sex (only for the model with overall participants), assessment centers, top 10 genetic principal components, and genotyping array.

Abbreviations: IVW, inverse-variance weighted; MR-Egger, Mendelian Randomization-Egger; WM, weighted median.

* MR-Egger intercept was used to detect the horizontal pleiotropy. *P*-value <0.05 indicated potential pleiotropy, which suggested exposure-associated genetic variants may influence the outcome through other pathways rather than through exposure.

After correcting for multiple comparison, *P*<0.05/3=0.017 was considered as significant.

Table S9. Association of genetically predicted short sleep duration with serum urate concentrations (µmol/L) using two-stage, IVW, MR-Egger, and WM methods.

| **Population and method** | **β (95% CI)** | ***P*-value** |
| --- | --- | --- |
| **Overall** |  |  |
| Two-stage | 2.91 (1.31 - 4.51) | < 0.001 |
| IVW | 3.93 (0.33 - 7.52) | 0.032 |
| MR-Egger | -3.64 (-22.70 - 15.43) | 0.712 |
| WM | -0.09 (-3.53 - 3.35) | 0.959 |
| MR Egger intercept* | \ | 0.439 |
|  |  |  |
| **Males** |  |  |
| Two-stage | 2.84 (0.29 - 5.38) | 0.029 |
| IVW | 3.26 (-2.05 - 8.58) | 0.229 |
| MR Egger | -11.80 (-34.58 - 10.98) | 0.321 |
| WM | 0.47 (-5.20 - 6.14) | 0.871 |
| MR Egger intercept* | \ | 0.197 |
|  |  |  |
| **Females** |  |  |
| Two-stage | 4.14 (2.03 - 6.24) | < 0.001 |
| IVW | 5.79 (0.58 - 11.00) | 0.029 |
| MR Egger | 2.20 (-21.51 - 25.92) | 0.857 |
| WM | 5.01 (0.37 - 9.65) | 0.034 |
| MR Egger intercept* | \ | 0.764 |

Adjusted for age, sex (only for the model with overall participants), assessment centers, top 10 genetic principal components, and genotyping array.

Abbreviations: IVW, inverse-variance weighted; MR Egger, Mendelian Randomization-Egger; WM, weighted median.

* MR-Egger intercept was used to detect the horizontal pleiotropy. *P*-value <0.05 indicated potential pleiotropy, which suggested exposure-associated genetic variants may influence the outcome through other pathways rather than through exposure.

After correcting for multiple comparison, *P*<0.05/3=0.017 was considered as significant.

Table S10. Association of genetically predicted long sleep duration with serum urate concentration (µmol/L) using two-stage, IVW, MR-Egger, and WM methods.

| **Population and method** | **β (95% CI)** | ***P*-value** |
| --- | --- | --- |
| **Overall** |  |  |
| Two-stage | -0.53 (-2.62 - 1.56) | 0.619 |
| IVW | -0.42 (-5.54 - 4.69) | 0.871 |
| MR-Egger | 2.63 (-15.03 - 20.30) | 0.785 |
| WM | -1.85 (-5.91 - 2.21) | 0.371 |
| MR-Egger intercept* | \ | 0.739 |
|  |  |  |
| **Males** |  |  |
| Two-stage | -2.26 (-5.09 - 0.57) | 0.117 |
| IVW | -2.77 (-10.80 - 5.26) | 0.499 |
| MR-Egger | 0.54 (-23.10 - 24.18) | 0.966 |
| WM | -4.58 (-10.24 - 1.09) | 0.113 |
| MR-Egger intercept* | \ | 0.780 |
|  |  |  |
| **Females** |  |  |
| Two-stage | -2.02 (-5.04 - 1.00) | 0.190 |
| IVW | -2.20 (-7.25 - 2.84) | 0.392 |
| MR-Egger | 12.53 (-8.82 - 33.89) | 0.333 |
| WM | -3.75 (-9.74 - 2.24) | 0.220 |
| MR-Egger intercept* | \ | 0.260 |

Adjusted for age, sex (only for the model with overall participants), assessment centers, top 10 genetic principal components, and genotyping array.

Abbreviations: IVW, inverse-variance weighted; MR-Egger, Mendelian Randomization-Egger; WM, weighted median.

* MR-Egger intercept was used to detect the horizontal pleiotropy. *P*-value <0.05 indicated potential pleiotropy, which suggested exposure-associated genetic variants may influence the outcome through other pathways rather than through exposure.

After correcting for multiple comparison, *P*<0.05/3=0.017 was considered as significant.


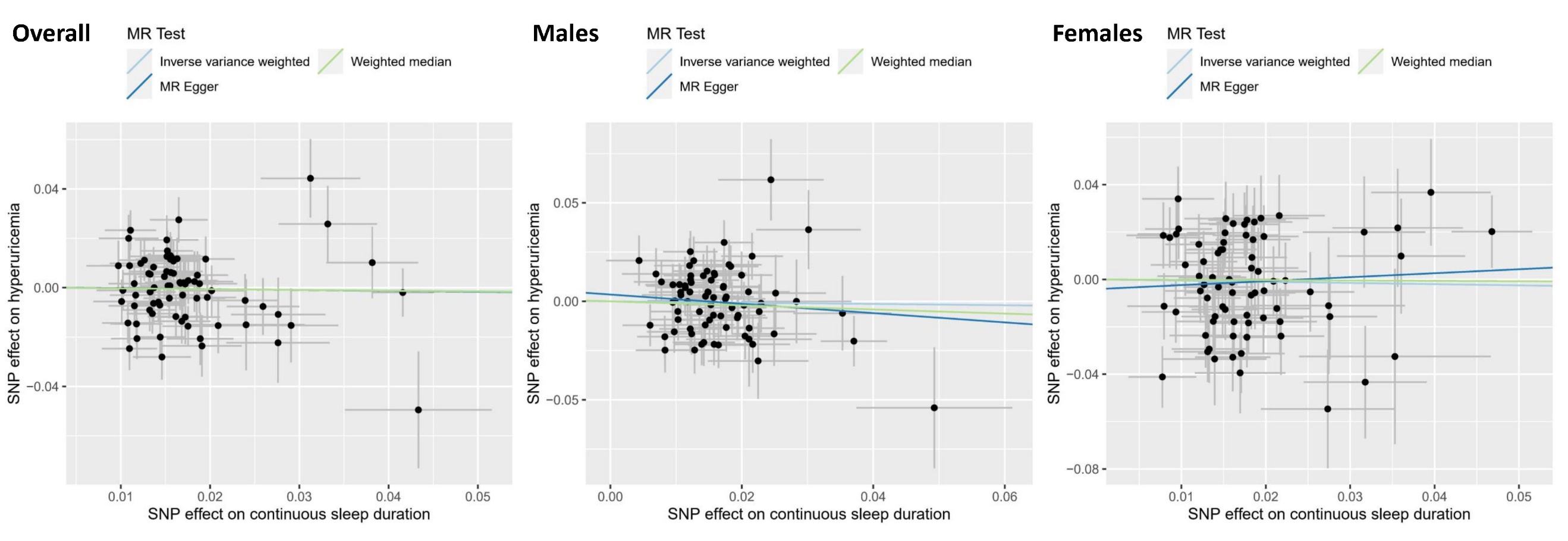


**Figure S1.** Scatter plot of association estimates for genetically predicted sleep duration and risk of hyperuricemia in overall participants, males, and females.

Mendelian randomization (MR) estimates from the inverse variance weighted, MR-Egger, and weighted median methods are provided. For each SNP, the effect and the standard error on the sleep duration and hyperuricemia are plotted.


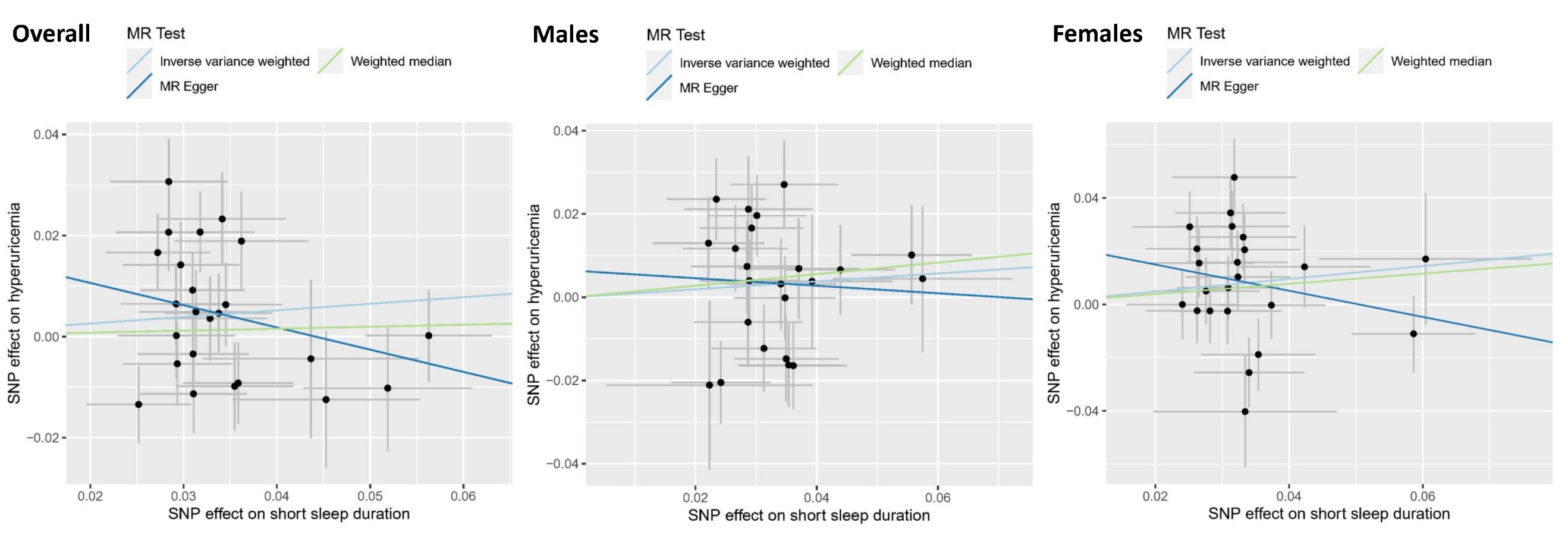


Figure S2. Scatter plot of association estimates for genetically predicted short sleep duration and risk of hyperuricemia.

Mendelian randomization (MR) estimates from the inverse variance weighted, MR-Egger, and weighted median methods are provided. For each SNP, the effect and the standard error on the short sleep duration and hyperuricemia are plotted.


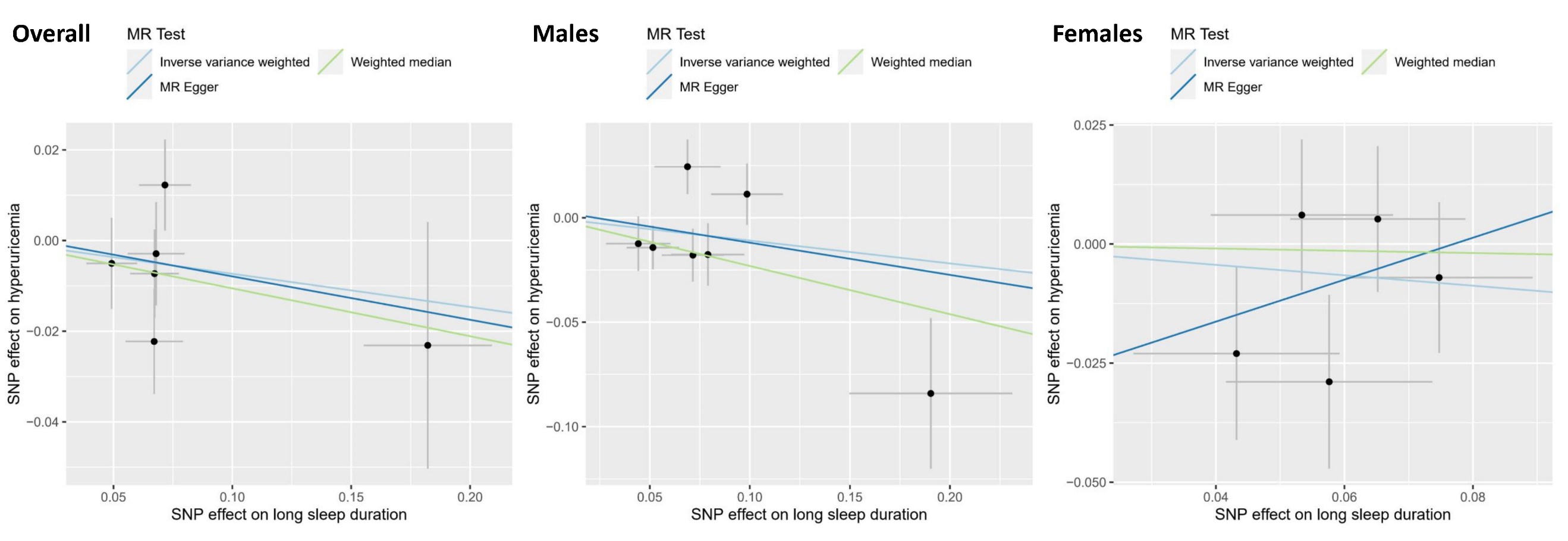


Figure S3. Scatter plot of association estimates for genetically predicted long sleep duration and risk of hyperuricemia.

Mendelian randomization (MR) estimates from the inverse variance weighted, MR-Egger, and weighted median methods are provided. For each SNP, the effect and the standard error on the long sleep duration and hyperuricemia are plotted.
